# A bilateral SPCN is elicited by to‐be‐memorized visual stimuli displayed along the vertical midline

**DOI:** 10.1111/psyp.14045

**Published:** 2022-03-22

**Authors:** Yanzhang Chen, Sabrina Brigadoi, Arianna Schiano Lomoriello, Pierre Jolicœur, Amour Simal, Shimin Fu, Valentina Baro, Roberto Dell'Acqua

**Affiliations:** ^1^ Department of Developmental Psychology University of Padova Padova Italy; ^2^ Department of Information Engineering University of Padova Padova Italy; ^3^ Section for Cognitive Systems, DTU Compute Technical University of Denmark Kgs. Lyngby Denmark; ^4^ Department of Psychology University of Montreal Montreal Quebec Canada; ^5^ Department of Psychology and Center for Brain and Cognitive Sciences Guangzhou University Guangzhou China; ^6^ Department of Neuroscience University of Padova Padova Italy; ^7^ Padova Neuroscience Center University of Padova Padova Italy

**Keywords:** cued visual search, ERPs, SPCN, visual working memory

## Abstract

We recently showed that deploying attention to target stimuli displayed along the vertical meridian elicits a bilateral N2pc, that we labeled N2pcb (*Psychophysiology*). Here we investigated whether a different component, the sustained posterior contralateral negativity (SPCN), shows the same property when a varying number of visual stimuli are displayed either laterally or on the vertical meridian. We displayed one or two cues that designated candidate targets to be detected in a search array that was displayed after a retention interval. The cues were either on the horizontal meridian or on the vertical meridian. When the cues were on the horizontal meridian, we observed an N2pc followed by an SPCN in their classic form, as negativity increments contralateral to the cues. As expected, SPCN amplitude was greater when two cues had to be memorized than when only one cue had to be memorized. When the cues were on the vertical meridian, we observed an N2pcb followed by a bilateral SPCN (or SPCNb). Critically, like SPCN, SPCNb amplitude was greater when two cues had to be memorized than when only one cue had to be memorized. A series of additional parametrical and topographical comparisons between N2pcb and SPCNb revealed similarities but also some important differences between these two components that we interpreted as evidence for their distinct neural sources.

## Abstract 2

We challenge the view that the SPCN ERP component cannot track the memory maintenance of objects displayed along the vertical meridian. Owing to the receptive fields of posterior neurons straddling on the intersection of the two visual hemifields, bilateral N2pc (N2pcb) and SPCN (SPCNb) activity can be detected using a cued visual search design.

## 1 INTRODUCTION

In order to identify visual stimuli of interest, we are required to scan our complex environment. In most cases, finding such objects does not seem to pose any insurmountable obstacle to our daily living. At the neural level, however, visual search involves a complex set of processes required to maintain a stable representation of the visual environment in spite of the massive changes of the retinal images caused by head and/or eye movements (e.g., Henderson, 2008; Hollingworth et al., 2008). Visuo‐spatial attention and visual working memory are said to play a crucial role in these processes, with visuo‐spatial attention often described as a filter set to individuate target stimuli, and visual working memory as a system optimized to maintain target information in a representational state amenable to further, higher‐level processing.

Studying visual attention and visual working memory in the lab using event‐related potentials (ERPs) has advanced our understanding of both these key aspects of human cognition, especially after the discovery that each of them is associated with a distinctive ERP signature. The ERP signature of the deployment of visuo‐spatial attention to candidate targets is the N2pc component (Eimer, 1996; Luck & Hillyard, 1994). N2pc is often studied in the context of visual search tasks. When a target is displayed laterally relative to fixation, N2pc manifests itself as a transient negativity enhancement usually unfolding in a 200–300 ms time‐window at parieto‐occipital sites (i.e., PO7/PO8) contralateral to the visual hemifield in which the target is displayed. The ERP signature of the active maintenance of a laterally displayed stimulus in visual working memory is the sustained posterior contralateral negativity component (SPCN; Jolicœur et al., 2008; alternatively named contralateral delay activity, or CDA, by Vogel & Machizawa, 2004; contralateral negative slow wave, or CNSW, by Klaver et al., 1999; contralateral search activity, or CSA, by Emrich et al., 2009). SPCN was initially explored using cued change detection tasks, in which subjects are typically cued to memorize objects displayed in either visual hemifield for later comparison with objects that can unpredictably remain the same or one of which can be changed. SPCN is often detected at the same recording sites as those used to observe N2pc (i.e., PO7/PO8) and, similarly to N2pc, manifests itself as a larger negativity contralateral to the visual hemifield in which target information is displayed. This surface similarity aside, SPCN onsets later (at about 400 ms^1^) and lasts substantially longer than N2pc, namely, as long as objects are retained in visual working memory (see Luria et al., 2016, for a comprehensive review). Furthermore, unlike N2pc,^2^ a distinctive feature of SPCN is that its amplitude increases as the number of objects to be retained in memory is increased, as long as this number does not exceed an individual's visual working memory capacity (Vogel & Machizawa, 2004), which averages to about 3 objects across individuals (Balaban et al., 2019; Cowan, 2001).

Source localization analyses of MEG recordings have localized the neural generators of the N2pc in the extra‐striate visual cortex, in the infero‐temporal cortex, with a possible early parietal contribution (Hopf et al., 2000, 2002, 2006; Jolicœur et al., 2011). MEG and fMRI recordings concur that the neural generators of SPCN are located in the parietal cortex, in the intra‐parietal sulcus in particular, and in more lateral/ventral regions also involved in the generation of N2pc activity (Becke et al., 2015; Brigadoi et al., 2017; Duma et al., 2019; Jolicœur et al., 2011; Naughtin et al., 2016; Robitaille et al., 2010; Todd & Marois, 2004; Xu & Chun, 2006). Although some uncertainty remains as to whether N2pc and SPCN have exactly the same or slightly different neural sources, it is important for the present purposes to note that the receptive fields of neurons located in the aforementioned regions and receiving inputs from foveal retinal receptors extend into the ipsilateral hemifield, a subset of them for as much as 2° of visual angle (Hubel & Wiesel, 1967; Nakamura et al., 2007; Papaioannou & Luck, 2020; Wandell et al., 2007; Zeki, 1993). As a result, visual input displayed along (or close to) the vertical meridian activates homologous neurons located in posterior regions of both hemispheres, and is therefore bilaterally represented in the posterior cortex.

Doro et al. (2020) have recently explored whether N2pc reflects this neuroanatomical organization of the receptive fields of neurons underpinning the selection and encoding phases of target information. Using a visual search task in which singleton or feature targets could be displayed laterally or aligned to the vertical meridian, we observed N2pc activity in its classical form, namely, as a larger negativity for contralateral relative to ipsilateral PO7/PO8 recording sites when targets were displayed laterally relative to the vertical meridian. Targets displayed along the vertical meridian elicited a bilateral negativity, that we quantified as the average activity detected at PO7 and PO8, that was undistinguishable from the contralateral negativity elicited by lateral targets. This pattern suggested that “midline” targets elicit a bilateral N2pc (or N2pcb; Doro et al., 2020; Monnier et al., 2020) that, like N2pc (e.g., Feldmann‐Wüstefeld & Schubö, 2015; Mazza et al., 2009), onsets earlier in singleton search than in feature search. Evidence for the supposed similarity between N2pc and N2pcb has also been reported by Monnier et al. (2020), who showed that N2pc and N2pcb share an additional property. It is now well established that the amplitude of N2pc is substantially reduced, sometimes even reversed in polarity, for lateral targets displayed above the horizontal meridian, that is, in the upper visual hemifield, compared to those displayed below the horizontal meridian, that is, in the lower visual hemifield (e.g., Bacigalupo & Luck, 2019; Luck et al., 1997). A likely explanation of this N2pc asymmetry refers to the neuroanatomical organization of the retinotopic topography in the posterior cortex. Stimuli in the lower visual field project to more dorsal regions of the posterior cortex, whereas stimuli in the upper visual field project to more ventral regions of the posterior cortex. Relative to ventral regions, dorsal regions are closer to the scalp, and this explains why N2pc can be more easily detected for stimuli in the lower visual field compared to stimuli in the upper visual field. In fact, using a singleton search design, Monnier et al. (2020) observed a fully‐fledged N2pc for lateral targets in the lower visual hemifield, and an N2pc polarity reversal for lateral targets in the upper visual hemifield (i.e., a contralateral positivity). Critically, an identical pattern was observed for N2pcb for midline targets when these targets were presented above versus below fixation, a result that was interpreted as suggesting a similarity of the neural sources of N2pc and N2pcb.

**FIGURE 1:**
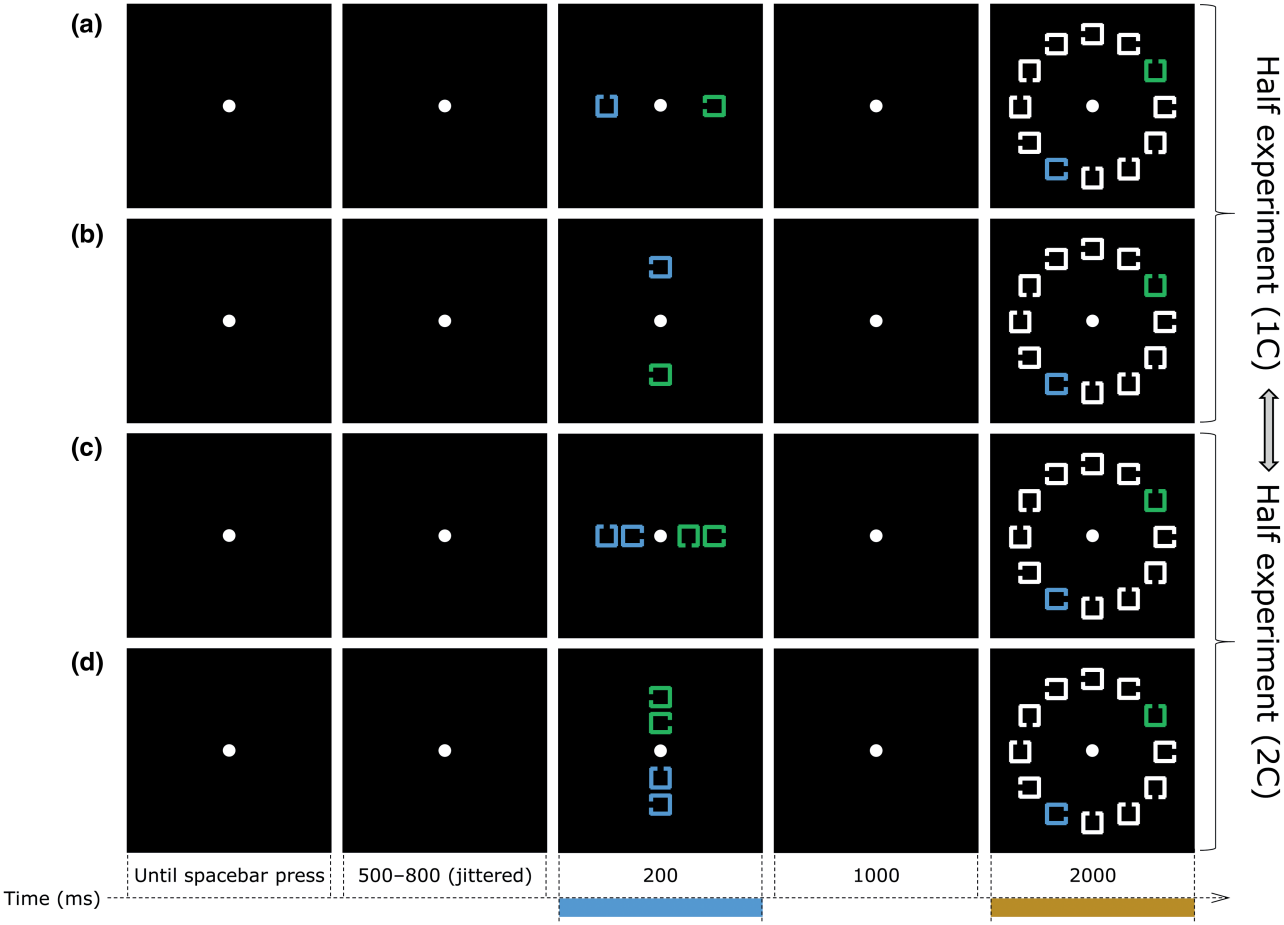
Sequence of events on four types of trials (a to d) in the present experiment showing the orthogonal combination of the number of items in the cue array (labeled here as 1C and 2C, as in trials a and b and in trials c and d, respectively) and the spatial arrangement of the cues, horizontal (as in trials a and c) or vertical (as in trials b and d). The stimuli in this figure are just approximately to scale with the stimuli displayed on the computer monitor. See section “2. Method” for details

The issue at stake in the present context is the lack of a test for SPCN conceptually analogous to those provided by Doro et al. (2020) and Monnier et al. (2020) for N2pc. Would a midline stimulus that must be retained in visual working memory elicit a bilateral SPCN (or SPCNb) of equal amplitude compared to the contralateral portion of the SPCN elicited by a lateral stimulus? Moreover, would SPCNb share with SPCN the peculiar property to scale in amplitude with the number of midline visual stimuli? Of course, given the overlap, or close proximity, of the neural generators of N2pc and SPCN activity, the expected answers to both these questions are in the positive. Perhaps, an issue that warrants close inspection in relation to the possible distinction of the neural sources of N2pc and SPCN would be to observe a different modulation of N2pc and SPCN as far as the vertical elevation of the visual stimuli is concerned. Would the amplitude of SPCN/SPCNb — similarly to the amplitude of N2pc/N2pcb — be reduced to nil, or even reversed in polarity, for stimuli displayed in the upper visual hemifield compared to SPCN/SPCNb elicited by stimuli displayed in the lower visual hemifield? To answer all these questions, we employed a cued visual search task akin to that of Carlisle et al. (2011), that is illustrated in Figure 1.

One or two colored squares (cues) with a gap on one side were displayed either on the horizontal meridian (left or right of fixation) or on the vertical meridian (above or below fixation) at the beginning of each trial. The cues of given color (e.g., green) indicated the candidate target(s), and subjects were instructed to memorize the position of the gap(s) for later search in an array composed of uniformly white distractor gapped squares, accompanied by a differently colored (blue) distractor in the opposite hemifield so as to avoid sensory imbalance. The task required first to select the candidate target(s) based on color, to keep the information about the gap position(s) in memory for a short interval (1 s), and finally to inspect a square of the same color as the cue(s) for a correspondence in gap position. The information needed to answer all the above questions were extracted from ERP activity time‐locked to the cue array onset. We estimated SPCN activity in the typical form, as the difference between ERP activity contralateral and ipsilateral to lateral cues recorded at PO7/PO8 electrodes. Similarly to how Doro et al. (2020) estimated N2pcb activity to midline search targets in their design, we estimated SPCNb activity as the difference between the bilateral ERP activity recorded at PO7/PO8 electrodes elicited by cues displayed on the vertical meridian and the ipsilateral ERP activity elicited by lateral cues. We expected to find clear SPCN activity during the retention of lateral cues that should be larger for two cues than for one cue, as reported by Carlisle et al. (2011). The new question asked here was whether we would find SPCNb activity of similar amplitude when the cues were presented aligned to the vertical midline. As argued in the foregoing introduction, this is what we expected, and in fact what we found.

## 2 METHOD

### 2.1 Participants

Twenty‐one students at the Guangzhou University (4 males; mean age = 23 years, *SD* = 2.4) took part in the present experiment after providing written informed consent. All participants had normal or corrected‐to‐normal visual acuity, and all reported normal color vision and no history of neurological disorders. The experiment was vetted by the local ethics committee.

### 2.2 Stimuli and procedure

An example of the stimuli and an illustration of the sequence of events on four trials in the experiment are shown in Figure 1. The stimuli were displayed on the black background (CIE: 0.312/0.329, 1.0 cd/m^2^) of a 17” CRT computer monitor with a refresh rate of 60 Hz, at a viewing distance of about 60 cm. The stimuli in the cue array (marked by the cyan bar on the timeline in Figure 1) were 2 or 4 equiluminant outlined squares (1.2° × 1.2°, 0.2° line thickness), colored in green (CIE: 0.278/0.393, 20 cd/m^2^) or in blue (CIE: 0.213/0.272, 20 cd/m^2^) with a gap (0.3°) on the left, right, top, or bottom side. When the cue array was composed of 2 gapped squares, each gapped square was displayed 3.5° to the left/right or above/below the center of the monitor. When the cue array was composed of 4 gapped squares, the 2 more eccentric gapped squares were presented 3.5° to the left/right or above/below the center of the monitor and the 2 less eccentric gapped squares were presented 1.8° to the left/right or above/below the center of the monitor. The stimuli in the search array (marked by the orange bar on the timeline in Figure 1) were 12 gapped squares identical in dimension to those composing the cue array, 10 of which were displayed in white (CIE: 0.313/0.329, 90 cd/m^2^), with the addition of two gapped squares, one blue and one green (same colors as the cues) always displayed laterally (i.e., left/right) on opposite sides relative to the center of the monitor. The stimuli in the search array were arranged along a notional circle of 5.8° in diameter and positioned in correspondence to the number locations on a clock face. With the exception of the positions aligned to the vertical meridian (i.e., the positions at 12 and 6 o'clock), all other positions on opposite sides relative to the center of the screen were equally likely to be occupied by the blue and green gapped squares.

Prior to the beginning of the experiment, each participant was informed about the task‐relevant color (i.e., either blue or green, counterbalanced across participants) designating cues and targets in the cue and search arrays, respectively. For each participant, the task‐relevant color was kept constant for the entire experiment. Each trial began with the presentation of a white fixation dot (0.4° × 0.4°) at the center of the monitor. Participants were instructed to maintain gaze on the fixation dot, avoiding head and/or eye movements until the end of the trial. Participants started each trial by pressing the spacebar using the thumb of the left or right hand. After the spacebar press, an interval of 500–800 ms (randomly jittered using a rectangular distribution) elapsed before the onset of the cue array, which was exposed for 200 ms. Participants had to memorize the position of the gap(s) of the cue(s) in the task relevant color. Participants had therefore to memorize the gap position of 1 cue (1C trials in Figure 1) or the gap positions of 2 cues (2C trials in Figure 1). The cues in the cue array could unpredictably and with equal probability be presented on the horizontal meridian (i.e., to the left/right of fixation) or on the vertical meridian (i.e., above/below fixation). The gap position(s) of the cue(s) in the cue array had to be memorized regardless of their spatial arrangement. The cue array was followed by an interval of 1000 ms, followed by the onset of the search array that was exposed for 2000 ms. On half of the trials, the search array contained a target, that is, a gapped square identical to the cue in 1C trials, or to either cue in 2C trials. On the other half of the trials, the target was absent. In the search array, the gap position of the (e.g., blue) cue never matched that of the (green) distractor. Participants were instructed to use the ‘L’ or ‘A’ of the computer keyboard (counterbalanced across participants) to indicate whether a target was present or absent, with equal emphasis on response speed and accuracy. Following the detection of the participant's response, the fixation dot disappeared and an inter‐trial interval of 1000 ms elapsed before the presentation of the fixation dot indicating the beginning of the next trial. Participants were informed that, during the intertrial interval, they were allowed to make eye blinks.

Participants performed a total of 10 blocks of 96 experimental trials each. Half of the participants started with 5 blocks of 1C trials, followed by 5 blocks of 2C trials. This order was reversed for the other half of the participants. Each series of 5 blocks was preceded by 18 to 24 1C or 2C practice trials, depending on which trials participants had to perform in the following blocks. Participants were informed they could take a short break between one block and the next.

### 2.3 EEG recording and pre‐processing

EEG activity was recorded continuously from 64 Ag/AgCl electrodes, positioned according to the 10–10 International system (Sharbrough et al., 1991), using a Neuroscan Curry 8 system (Compumedics USA, Charlotte, NC, USA) set in AC mode and using an electrode located between FPz and Fz as ground. Vertical electrooculogram (VEOG) was recorded from two electrodes positioned 1.5 cm above and below the left eye. Horizontal electrooculogram (HEOG) was recorded from two electrodes positioned on the outer canthi of both eyes. EEG, VEOG, and HOEG signals were band‐pass filtered between 0.01 and 30 Hz and digitized at a sampling rate of 1000 Hz. EEG activity was referenced online to an electrode located approximately 1.5 cm posterior to Cz and re‐referenced offline to the average value of the left and right mastoids. Continuous EEG was then segmented into 1800 ms long epochs, starting 200 ms before the onset of the cue array and ending 400 ms after search array presentation. EEG epochs were baseline corrected by using the average activity in the time interval − 200–0 ms relative to onset of the cue array. Artifact detection was performed using a 200 ms sliding window peak‐to‐peak analysis, with a threshold of 80 μV for the EOG electrodes and 100 μV for all other channels (Vaskevich et al., 2021). Participants with more than 30% of rejected trials were excluded from subsequent analyses. After excluding trials associated with eye movements and those associated with an incorrect response in the visual search task, independent component analysis (ICA) was applied to correct EEG activity for residual eyeblinks and eye movements (Jung et al., 1997; see Drisdelle et al., 2017, for a detailed description of the method and validation for use with lateralized ERP components). EEG epochs were then averaged to generate ERPs for each set of 1C and 2C trials. For laterally displayed cues, contralateral ERPs were generated by averaging EEG epochs recorded at PO7 on trials with cues displayed to the right of fixation and EEG epochs recorded at PO8 on trials with cues displayed to the left of fixation. Ipsilateral ERPs were generated using the opposite electrode‐side pairings. For cues displayed along the vertical midline, a bilateral ERP was generated by averaging EEG epochs recorded at PO7 and PO8. The mean amplitude of the N2pc and of the SPCN elicited by lateral cues was computed by subtracting the ipsilateral activity from the contralateral activity in a 200–300 ms interval and in a 360–1100 ms interval, respectively. As in Doro et al. (2020), the mean amplitude of the N2pcb and of the SPCNb elicited by midline cues was computed by subtracting the ipsilateral activity elicited by lateral cues from the bilateral activity elicited by midline cues in the same time‐windows as those considered for N2pc and SPCN amplitude estimation.

EEG data in the N2pc/N2pcb and SPCN/SPCNb time‐windows were transformed to scalp current density (SCD) topographic maps using a spherical spline surface Laplacian (order of the splines = 4, regularization parameter λ = 1e‐5, conductivity of the skin = 0.33 S/m) (Perrin et al., 1989). We opted for SCD maps because the SCD approach provides a sharper topography compared to spline‐interpolated maps of voltage intensity by reducing blurring effects of volume conduction on the scalp‐recorded EEG voltage signal (Pernier et al., 1988). In particular, SCD maps provide reference‐free mapping of scalp‐recorded electrical activity, thus rendering ERP polarity unambiguous. The SCD approach to scalp topography does not make any assumptions about the neuroanatomy or about the number, orientation, or independence of the underlying neuronal generators. The sign of these estimates directly reflects the direction of the global radial currents underlying the EEG topography, with positive values representing current flow from the brain towards the scalp, and negative values representing current flow from the scalp into the brain.

All statistical analyses were performed with R (R Development Core Team, 2017), using the ezANOVA function of the ‘ez’ package (Lawrence, 2011) and the anovaBF/ttestBF function of the “BayesFactor” package (Rouder & Morey, 2012), which includes the Jeffreys‐Zellner‐Siow (JZS) default prior on effect sizes (Rouder et al., 2012). Greenhouse–Geisser correction for non‐sphericity was applied when appropriate (Jennings & Wood, 1976), and all comparisons via *t*‐test were Bonferroni‐corrected.

## 3 RESULTS

Two participants were discarded from all analyses for an excessive percentage of trials rejected because of eye movements (54.1% and 65.4%, respectively). For the remaining nineteen participants, the mean percentage of rejected trials was 10.9% (range = 0.6%–29.7%).

### 3.1 Behavior

**FIGURE 2:**
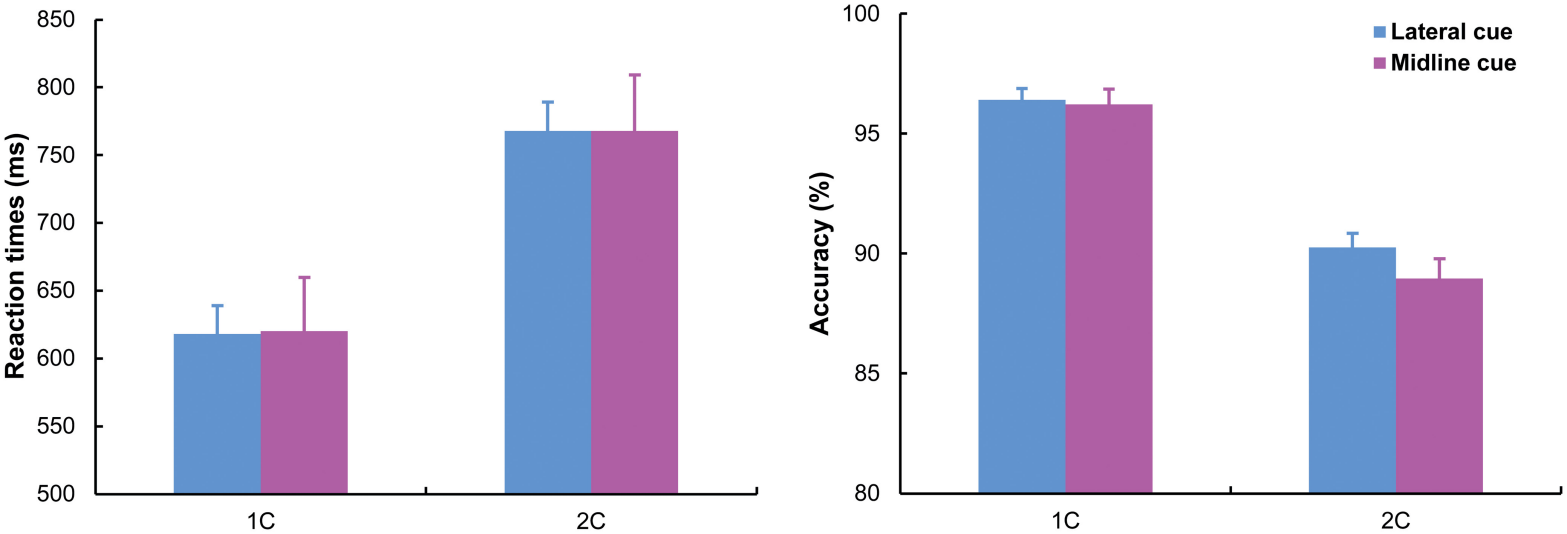
Mean RT (left panel) and mean percentage of correct responses (right panel) in the visual search task plotted as a function of memory load (1C vs. 2C) and cue position (lateral vs. midline). Error bars represent standard error of the mean

Reaction times (RTs) recorded on trials associated with an incorrect response and/or RTs exceeding three standard deviations above/below individual mean RT (1.6%) were excluded from analysis. A summary of the behavioral results in the visual search task is illustrated in Figure 2.

Mean RTs and the mean percentage of correct responses were submitted to a 2 × 2 ANOVA considering memory load (1C vs. 2C) and cue position (lateral vs. midline) as within‐subject factors. RTs were generally shorter on 1C than 2C trials (*F*(1, 18) = 35.3, *p* < .001, $\eta_{p}^{2}$ = .663). RTs were unaffected by cue position, or by the interaction between cue position and memory load (max *F* = 0.4, min *p* = .6). Participants were more accurate on 1C than 2C trials (*F*(1, 18) = 100.8, *p* < .001, $\eta_{p}^{2}$ = .848), and more accurate with lateral than midline cues (*F*(1, 18) = 5.7, *p* = .028, $\eta_{p}^{2}$ = .242). The interaction between cue position and memory load was significant (*F*(1, 18) = 5.76, *p* = .027, $\eta_{p}^{2}$ = .242), indicating that participants were more accurate with lateral than midline cues only in 2C trials (90.3% vs. 89.0%, respectively; *t*(18) = −3.3, *p* = .004; 1C trials: 96.4% vs. 96.2%, respectively; *t*(18) = −0.5, *p* = .641). It is not immediately clear the source of this slight drop in accuracy when participants performed the visual search task (do note: for targets always displayed laterally in the search array) when two cues were displayed on the vertical midline. One speculative explanation refers to the detrimental effect on response accuracy of the neural overlap of a unilaterally represented lateral target with bilaterally represented midline cues (Cohen et al., 2014). Such overlap affected all trials with midline cues — the more so when two cues had to be processed rather than one cue — but only half of trials with lateral cues and target displayed in the same hemifield.

Mean RTs and the mean percentage of correct responses for midline cues were submitted to a further 2 × 2 repeated measures ANOVA considering visual hemifield (upper vs. lower) and memory load (1C vs. 2C) as within‐subject factors. The analysis did not detect any significant effects (max *F* = 2.4, min *p* = .141).

### 3.2 Event‐related potentials

#### 3.2.1 SPCN and SPCNb

**FIGURE 3:**
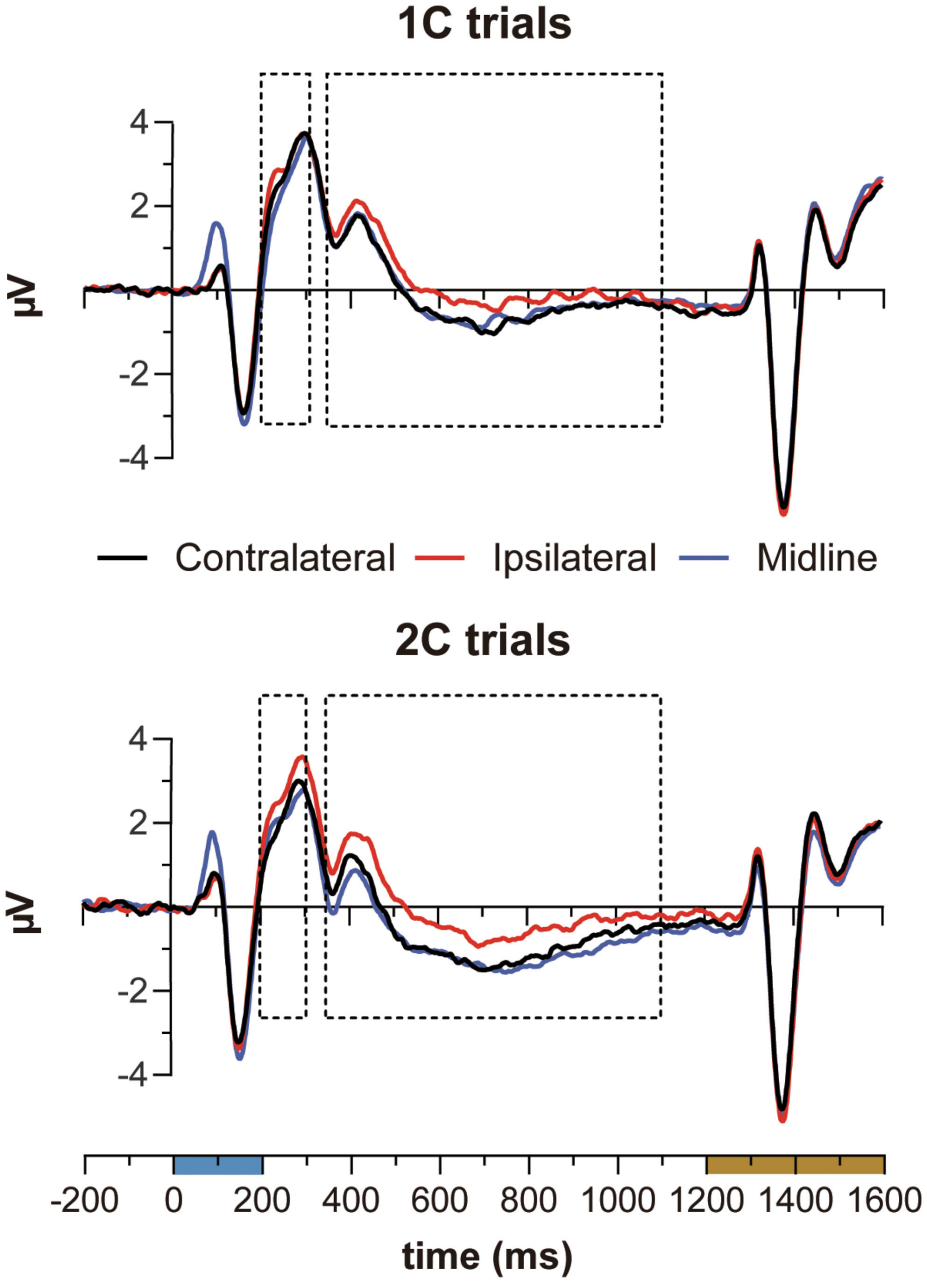
ERPs elicited at electrodes PO7/8 on 1C (top) and 2C (bottom) trials. Color bars on the timeline indicate the exposure duration of the cue array (cyan) and of the search array (dark orange). The areas delimited by the dashed‐line rectangles in both graphs indicate the time‐windows considered for ERP amplitude estimation. Negative is plotted down in this and following ERP graphs

Figure 3 illustrates grand‐average contralateral and ipsilateral ERP waveforms recorded at PO7/8 elicited by lateral cues and ERP waveforms elicited by midline cues generated by averaging EEG epochs recorded at the same recording sites.

**FIGURE 4:**
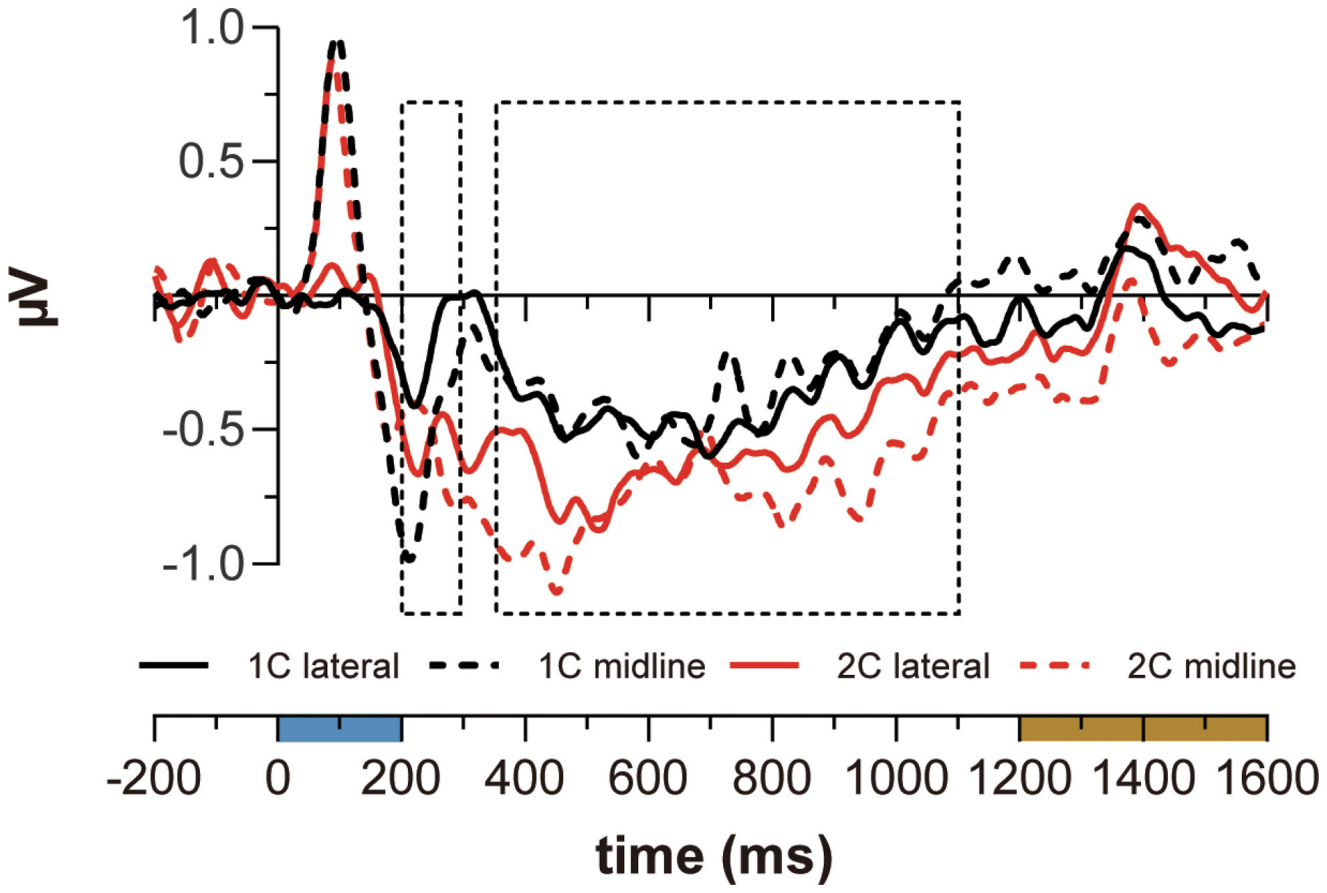
Difference ERPs on 1C and 2C trials. The areas delimited by the dashed‐line rectangles in the graph indicate the time‐windows considered for ERP amplitudes estimation. SPCN activity is represented by solid‐line ERP functions and SPCNb by dashed‐line ERP functions. SPCN and SPCNb activity recorded on 1C trials is represented by black ERP functions and SPCN/SPCNb activity recorded on 2C trials is represented by red ERP functions. ERP functions were low‐pass filtered at 15 Hz for visualization purposes

Visual inspection of Figure 3 makes apparent — in the SPCN/SPCNb time‐window (360–1100 ms) — the substantial overlap of ERPs contralateral to lateral cues (black functions in Figure 3) and the ERPs to midline cues (blue functions in Figure 3) on both 1C and 2C trials. Furthermore, the comparison between both contralateral and midline ERPs and ipsilateral ERPs to lateral cues (red functions in Figure 3) suggests that both SPCN and SPCNb increased in amplitude as the number of cues was increased. This is more evident in Figure 4, where difference ERPs are plotted. Recall that the amplitude of SPCN was calculated in the standard way by subtracting ipsilateral from contralateral ERP activity elicited by lateral cues. The amplitude of SPCNb was calculated by subtracting ipsilateral ERPs for lateral cues from the average of ERPs at PO7 and PO8 for midline cues.

These observations were corroborated by statistical analysis. The amplitude values recorded in the SPCN/SPCNb time‐window were first separately submitted to *t*‐test to determine whether they differed from 0 μV. SPCN amplitude was significant for both 1C (−0.40 μV; *t*(18) = −4.5, *p* < .001) and 2C trials (−0.60 μV; *t*(18) = −5.3, *p* < .001). Similarly, SPCNb amplitude was significant for both 1C (−0.30 μV; *t*(18) = −2.8, *p* = .012) and 2C trials (−0.62 μV; *t*(18) = −3.8, *p* = .001).

These amplitude values were then submitted to a 2 × 2 ANOVA with memory load (1C vs. 2C) and cue position (lateral vs. midline) as within‐subject factors. The analysis detected a main effect of memory load (*F*(1, 18) = 8.9, *p* = .008, $\eta_{p}^{2}$= .330), and no other factor effects (max *F* = 0.6; min *p* = .5). Given that the null effects of cue position and of an interaction between cue position and memory load were critical to support our hypothesis of an amplitude equivalence of SPCN and SPCNb, Bayes factors (*BF*_01_) were estimated using mixed‐effect models in which participants were treated as an additional random factor. A Type 2 approach was adopted to not violate the principle of marginality (Nelder, 1977). The *BF*_01_ parameter approximates the probability that a given null effect or interaction is truly absent relative to the alternative hypothesis of the presence of such effects. A *BF*_01_ value higher than 1 is usually taken to imply that the probability of the (possibly undetected) presence of such effects in the statistical comparison between SPCN and SPCNb is minimal/anecdotal. The *BF*_01_ was 4.08 for the effect of cue position and 2.72 for the interaction of cue position and memory load. These results provide critical support for the statistical equivalence of SPCN and SPCNb amplitudes on 1C and 2C trials.

#### 3.2.2 N2pc and N2pcb

The present design allowed us to test whether the results of Doro et al. (2020) with reference to the amplitude equivalence of N2pc (lateral targets) and N2pcb (midline targets) could be replicated. The ERP results illustrated in Figures 3 and 4 do suggest that this might be the case.^3^ As for SPCN/SPCNb, the amplitude values recorded in the N2pc/N2pcb time‐window (see Figure 4) were first separately submitted to *t*‐test to inspect whether each of these values differed from 0 μV. N2pc amplitude was not significantly different from 0 μV in 1C trials (−0.20 μV; *t*(18) = −1.5, *p* = .15), but was clearly present in 2C trials (−0.56 μV; *t*(18) = −3.7, *p* = .002). N2pcb amplitude was significant in both 1C trials (−0.60 μV; *t*(18) = −4.1, *p* < .001) and 2C trials (−0.57 μV; *t*(18) = −2.5, *p* = .02).

These amplitude values were then submitted to a 2 × 2 ANOVA with memory load (1C vs. 2C) and cue position (lateral vs. midline) as within‐subject factors. The analysis detected a marginal interaction between memory load and cue position (*F*(1, 18) = 4.3, *p* = .052, $\eta_{p}^{2}$= .194), which was most likely driven by the smaller N2pc in 1C trials. Further planned comparisons showed that the amplitude of N2pcb was greater than that of N2pc on 1C trials (−0.60 μV vs. −0.20 μV; *t*(18) = 2.4, *p* = .026), whereas no amplitude difference between N2pc and N2pcb was found in 2C trials (−0.56 μV vs. −0.57 μV; *t*(18) = −0.04, *p* = .968). Although we do not have an explanation for the minimal N2pc activity (*vis‐a‐vis* the clear presence of N2pcb activity) on 1C trials, when collectively taken these results support and reinforce Doro et al. (2020) hypothesis of the existence of N2pcb activity elicited by midline cues. Visual inspection of the results illustrated in Figure 2 by Carlisle et al. (2011; Experiment 1, p. 9317) suggests that even in their case N2pc for one lateral cue was smaller in amplitude than N2pc for two lateral cues. Given it was outside the scope of their work, however, N2pc amplitude was not quantified and/or analyzed by Carlisle et al. (2011), and future work may profitably be addressed to investigate this interesting analogy between the present and Carlisle's et al. results.

#### 3.2.3 N2pcb and SPCNb for upper and lower visual hemifield cues

On the hypothesis of similar sources of N2pcb and SPCNb — and, indirectly, of N2pc and SPCN — one would expect SPCNb to share with N2pcb the property described by Monnier et al. (2020) to be fully‐fledged in response to task‐relevant information displayed in the lower visual hemifield and absent, or even reversed in polarity, in response to task‐relevant information displayed in the upper visual hemifield.

**FIGURE 5:**
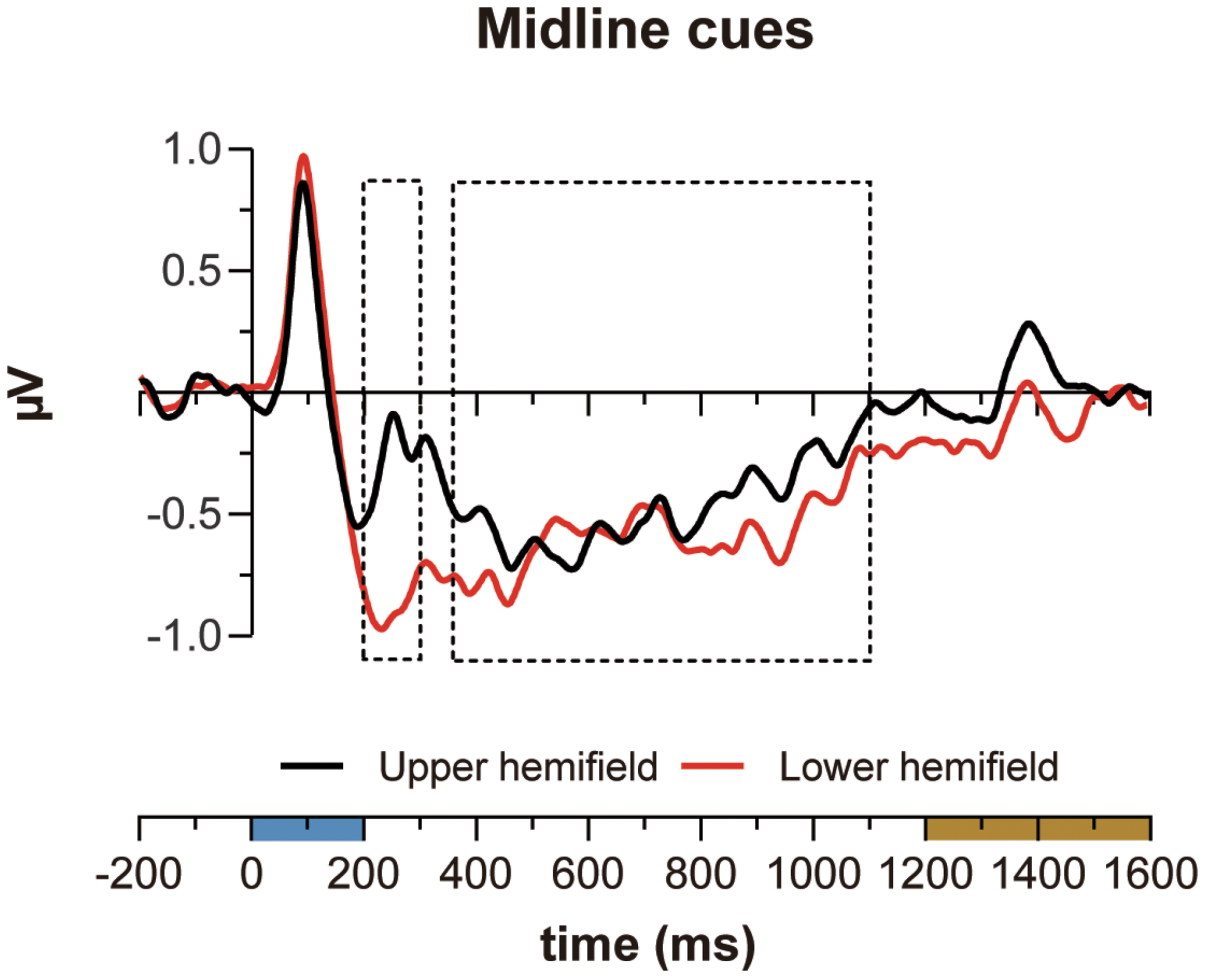
Difference ERPs for midline cues presented in the upper (black function) and lower (red function) hemifields. The area indicated by the dashed‐line rectangles in the graph represents the time window considered for ERP amplitude analyses. ERP functions were low‐pass filtered at 15 Hz for visualization purposes

The difference ERP waveforms, collapsed across 1C and 2C trials, elicited by midline cues displayed in the upper and lower visual hemifields are shown in Figure 5.

As Figure 5 suggests, N2pcb amplitude variations were strongly modulated by cue vertical elevation, as reported by Monnier et al. (2020). N2pcb was clearly larger for cues displayed in the lower visual hemifield and basically absent for cues displayed in the upper visual hemifield (−0.90 μV vs. −0.27 μV, respectively; *t*(18) = 4.9, *p* < .001). In contrast, SPCNb for cues displayed in the upper visual hemifield, though seemingly reduced in amplitude compared to SPCNb for cues displayed in the lower visual hemifield, was nonetheless clearly evident (−0.45 μV vs. −0.47 μV, respectively; *t*(18) = −0.2, *p* = .819). The amplitude values of N2pcb and SPCNb were submitted to a 2 × 2 ANOVA with visual hemifield (upper vs. lower) and component (N2pcb vs. SPCNb) as within‐subject factors. The analysis revealed a significantly larger amplitude for lower than upper visual hemifield (*F*(1, 18) = 7.4, *p* = .014, $\eta_{p}^{2}$= .190) and, more importantly, a significant interaction between component and visual hemifield (*F*(1, 18) = 72.8, *p* < .001, $\eta_{p}^{2}$= .802). Planned comparisons confirmed that, for cues displayed in the upper visual hemifield, N2pcb amplitude did not differ from 0 μV (−0.27 μV; *t*(18) = −1.5, *p* = .141) whereas SPCNb amplitude did (−0.45 μV; *t*(18) = −3.1, *p* = .007). In contrast, N2pcb and SPCNb were both significant for lower‐hemifield targets (−0.90 and − .47 μV; *t*(18) = −5.0, *p* < .001 and *t*(18) = −3.8, *p* = .001, respectively). To ensure the only difference between cues displayed in the upper and lower visual hemifield was reflected in the N2pcb, we computed also the P1 component, estimated in a 60–120 ms time window. There was no significant difference in P1 activity for upper vs. lower hemifield midline cues (*t*(18) = −1.7, *p* = .113).

**FIGURE 6:**
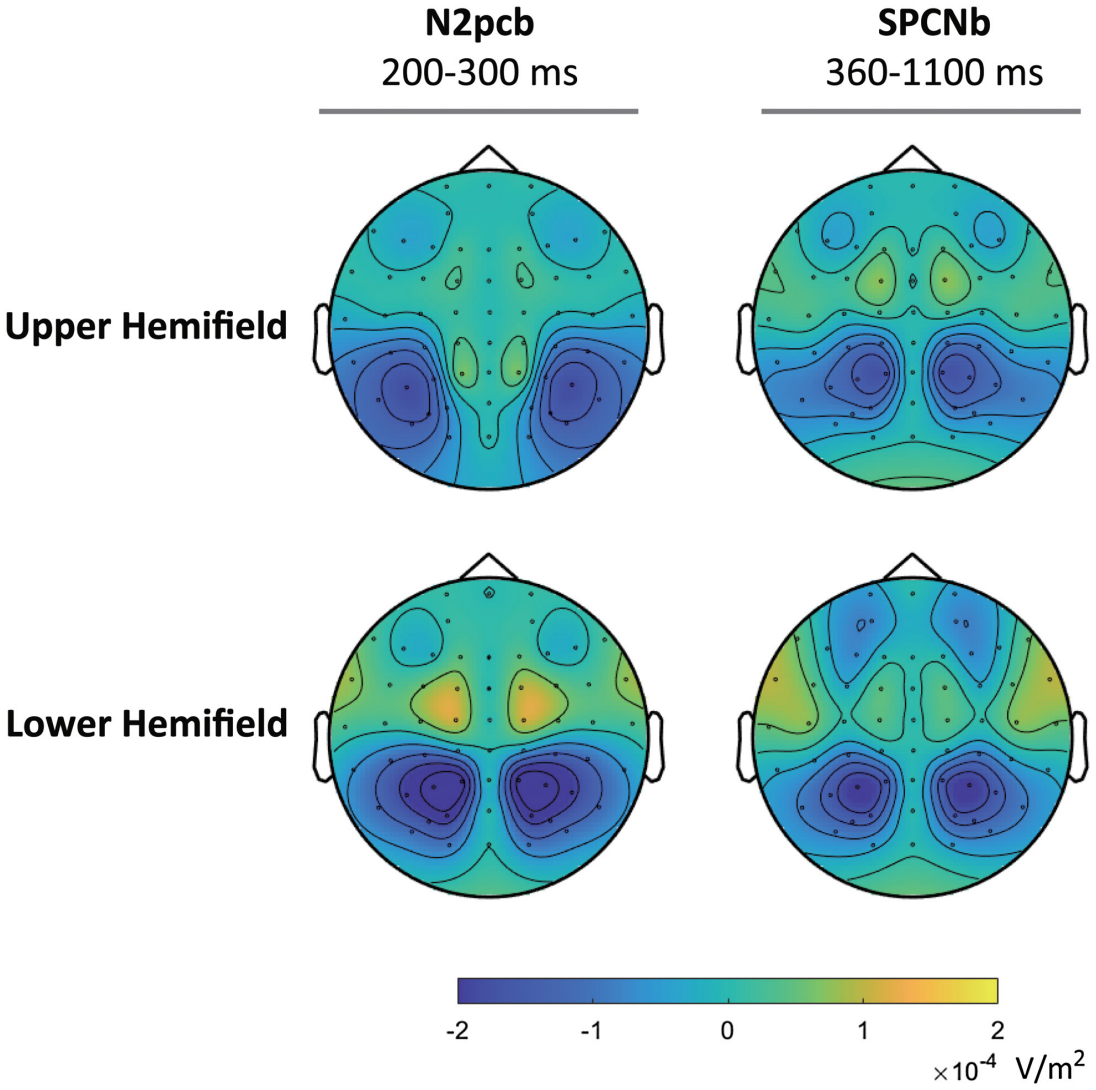
Scalp current density (SCD) maps of N2pcb (left) and SPCNb (right) difference ERPs for midline cues presented in the upper and lower hemifields. The components are plotted mirrored in both the hemiscalps

Hints to a possible cause of the different behavior of N2pcb and SPCNb in response to cues displayed in the upper and lower visual hemifields can be inferred from the topographical maps reported in Figure 6.

By comparing the peak of the current densities elicited by cues displayed in the upper visual hemifield, the impression is that the density peak of N2pcb activity is slightly more lateral/ventral in comparison to SPCNb, whose density peak is more dorsal and closer to the mid‐scalp. As argued in the Introduction, EEG signals originating from dorsal regions are easier to detect because closer to the scalp, and this may explain why SPCNb activity, though reduced in amplitude, could still be detected whereas N2pc activity was abolished for cues displayed in the upper visual hemifield. Given however the notoriously complex nature of the relationship between the scalp distribution of EEG signal and the brain location of its neural source(s), this explanation must be taken with caution. It is nonetheless worthy of mention that the present topographical results dovetail nicely with source localization analyses of MEG signal reported by Becke et al. (2015), Hopf et al. (2000, 2002, 2006), Jolicœur et al. (2011), and Robitaille et al. (2010) that converged to locate the source of SPCN activity in dorso‐parietal cortical regions and the source of N2pc activity in ventro‐lateral cortical regions.

## 4 DISCUSSION

To summarize, we showed that to‐be‐memorized visual cues displayed along the vertical midline elicited a bilateral SPCN, or SPCNb, whose amplitude was identical to the SPCN elicited by visual cues displayed laterally relative to the vertical meridian. Confirming a prototypical property of SPCN, both SPCN and SPCNb increased in amplitude as the number of cues was increased from one (on 1C trials) to two (on 2C trials). With the due caution given we did not show a plateau for SPCNb for supra‐capacity memory loads, we argue that this pattern of results indicates that a) SPCNb does exist as a distinguishable ERP component and that b) SPCNb reacts to variations in visual working memory load in a similar way to SPCN. Behaviorally, RTs were faster and accuracy was higher when visual search was guided by one cue than two cues, but apart from a slight drop in accuracy when search was guided by two midline cues, search performance was generally unaffected by the spatial arrangement of the cues in the leading cue array. Furthermore, we compared amplitude modulations of SPCNb and N2pcb as a function of the vertical elevation of midline cues, and discovered a dissociation between these two ERP components. Like N2pc, N2pcb was absent when midline cues were displayed in the upper visual hemifield (i.e., above fixation), and was present and particularly pronounced when midline cues were displayed in the lower visual hemifield (i.e., below fixation) (Bacigalupo & Luck, 2019; Doro et al., 2020; Luck et al., 1997; Monnier et al., 2020). In contrast, when midline cues were displayed in the upper visual field, SPCNb was slightly reduced in amplitude — but still clearly present — relative to SPCNb for midline cues displayed in the lower visual field. This finding was complemented by a comparison of N2pcb and SPCNb based on SCD topography, which suggested a more dorsal distribution of SPCNb activity and a more latero‐ventral distribution of N2pcb activity. We interpret this pattern of results as consistent with results from MEG explorations of N2pc and SPCN (Becke et al., 2015; Hopf et al., 2000, 2002, 2006; Jolicœur et al., 2011; Robitaille et al., 2010), that pointed to a prominent involvement of the lateral occipital complex (LOC) and infero‐temporal (IT) cortex in the generation of N2pc/N2pcb activity and of the intra‐parietal sulcus (IPS) in the generation of SPCN/SPCNb activity (see, for fMRI evidence, Brigadoi et al., 2017; Duma et al., 2019; Jolicœur et al., 2011; Naughtin et al., 2016; Robitaille et al., 2010; Todd & Marois, 2004; Xu & Chun, 2006).

Two issues deserve a comment with reference to the present ERP findings. One issue pertains to a possible methodological concern related to the fact that we calculated N2pc and SPCN amplitude for laterally displayed visual cues by subtracting (ipsilateral from contralateral) ERP activity, that is, ERP activity that, though from different electrodes, was recorded on the same trials. N2pcb and SPCNb amplitude for midline cues was calculated using a different approach, by subtracting ERP activity that was recorded on different trials (ipsilateral to lateral cues from bilaterally averaged to midline cues). As we have already claimed in Doro et al. (2020), this choice relies on the assumption that ipsilateral activity for laterally displayed stimuli is relatively invariant to factors' manipulations that exert modulatory effects on N2pc/N2pcb and SPCN/SPCNb amplitude (and latency), implicating that such effects are reflected in modulations of the contralateral portion of these ERP components. As far as N2pc/N2pcb activity is concerned, we provided a comprehensive overview of studies supporting this assumption in Doro et al. (2020), in all of which ipsilateral activity to lateral stimuli in the N2pc time‐window remained largely invariant across a number of manipulations affecting the contralateral portion of N2pc. This was the case for manipulations affecting target color (Luck et al., 2006), target vs. nontarget feature selection (Luck & Hillyard, 1994), target position relative to the horizontal midline (Luck et al., 1997; Perron et al., 2009), target numerosity (Benavides‐Varela et al., 2018; Mazza & Caramazza, 2011), and target selection difficulty (Luck et al., 1997).

As far as SPCN/SPCNb activity is concerned, we felt even more confident in treating ipsilateral activity as a common baseline for SPCN and SPCNb amplitude calculation based on the flood of work showing that ipsilateral activity is largely unaffected by manipulations of the number of to‐be‐memorized visual stimuli in the paradigm used here (cued visual search paradigms; Carlisle et al., 2011), as well as in other paradigms like change detection (see Luria et al., 2016, for a comprehensive and detailed overview), multiple object tracking (MOT; when no moving objects crossed the vertical midline, see below; Drew et al., 2013; Drew & Vogel, 2008), and in feature conjunction/grouping paradigms (Luria & Vogel, 2011). To our knowledge, the only exception to the ipsilateral activity invariance in the SPCN/SPCNb time‐range is the tendency of ipsilateral activity to become progressively more negative when the retention interval (i.e., the time elapsing from the offset of to‐be‐memorized visual stimuli to the onset of the event probing visual working memory efficiency) is longer than 1 s (McCollough et al., 2007). Our retention interval was 1 s, and the ipsilateral ERP activity plotted in Figure 3 in the selected time‐window (360–900 ms from the *onset* of the cue array) did not appear to be deflected toward the negative polarity to such an extent as to determine SPCN/SPCNb amplitude.

Note however that the assumption of invariance of ipsilateral activity is not necessarily in opposition to the hypothesis that such activity reflects some form of suppression/inhibition of ipsilateral stimuli, as originally put forth by Hickey et al. (2009) for N2pc. An equally plausible stance — which is also in line with current empirical evidence on the role of suppression during visual encoding — is that stimuli falling in the ipsilateral visual hemifield are just suppressed as a single chunk, irrespective of their number and other physical attributes, provided no feature overlap (Kiss et al., 2008) or a particularly pronounced salience disparity is present between target and distractors (Gaspar et al., 2016; Gaspelin & Luck, 2018, 2019).

In the Introduction, we mentioned that the receptive fields of extrastriate visual neurons extend into the ipsilateral visual hemifield for as much as 2°. This calls for a clarification concerning the horizontal extension of the area covered by overlapping receptive fields of visual neurons located in each cerebral hemisphere. Certainly, this bilaterally represented area includes the vertical midline, but its lateral extension has been shown to vary considerably based on participants' expectation. One of the most convincing demonstration of this has been provided by Drew et al. (2014), who instructed participants to first select a static object that was temporarily cued by a color and then to covertly track it when the object started moving. SPCN component, whose amplitude correlates with the number of objects tracked at any one time in the contralateral visual hemifield, was monitored in order to understand how an object moving in a lateral direction and crossing the vertical midline was represented in the posterior cerebral hemispheres. In one of their experiments, one laterally moving object eventually crossed the vertical midline on each trial. The SPCN recorded from the hemisphere contralateral to the starting position of this moving object decreased in amplitude (i.e., stopped tracking the moving object) only after the object was 2° past the vertical midline, whereas SPCN activity recorded from the ipsilateral hemisphere started to increase in amplitude (i.e., started to track the moving object) 1.2° before the object crossed the vertical midline. Interestingly, in another experiment, the event of a lateral object crossing the vertical meridian occurred only on a random 25% of trials. In this condition, the SPCN recorded from the hemisphere contralateral to the starting position of the moving object showed the same response as that in the previous experiment, but signs of SPCN activity in the ipsilateral hemispheres started to be detected when the object was almost 3° *past* the vertical midline. The interpretation of these results offered by the authors was one according to which the extension of the area of overlapping activity of the cerebral hemispheres is not structurally determined, but changes dynamically as a function of the participants' attentional set. These results are of clear relevance in the present context. Although in our paradigm midline cue(s) were displayed on a random 50% of trials, they were perfectly aligned to the vertical midline, a segment of the visual field that is structurally bound to be always represented by both cerebral hemispheres. However, it is important to underline that our expectations and/or attentional set can dynamically change the way in which the integration of separate visual hemifields occurs and to what extent, a property that we could not capture in the present study and that certainly warrants further investigation. Incidentally, one neural model that provides a plausible explanation of how dynamic changes in the size of receptive fields may be possible is that of Lamme and Roelfsema (2000), who proposed that one effect of reentrant activity from frontal to more posterior regions is to expand local sensory circuits by coaxing visual neurons that did not contribute to the initial feedforward volley of activation upon stimulus presentation.

The hypothesis of SPCNb as a bilateral SPCN elicited by midline memoranda invites a consideration with reference to prior reports describing a bilaterally recorded “negative slow wave” (NSW; Ruchkin et al., 1995; Ruchkin et al., 1992) in response to central and bilateral visual memoranda. Of import, like SPCN, NSW has been shown to increase in amplitude as the number of items in visual working memory is increased, and to reach a plateau in response to supra‐capacity memory loads (e.g., Diaz et al., 2021; Fukuda et al., 2015). These findings seem to legitimate the inference that NSW and SPCN may be one and the same ERP component. To our knowledge, only one study has parametrically addressed the issue of the comparability between SPCN and NSW. Feldmann‐Wüstefeld (2021) concomitantly estimated NSW and SPCN activity using a design in which the total number (set size) and the numerical imbalance (net load) of to‐be‐memorized colored circles was systematically varied while keeping the overall stimulation sensorially balanced by varying the spatial distribution of to‐be‐ignored colored squares. SPCN activity — recorded at PO7/PO8 electrodes from trials in which the colored circles were unevenly distributed between hemifields — manifested itself as a negativity imbalance (i.e., more negative contralateral to the side where more circles were displayed) modulated by variations in net load. NSW activity — recorded at the same electrodes as those used to detect SPCN — manifested itself as a bilateral increment in negativity modulated by variations in set size. The functional similarity between NSW and SPCN was inferred in this study based on findings showing that both these ERP components correlated with individual Cowan's K estimates of visual working memory capacity. Note that on the hypothesis of an exact equivalence between SPCNb and NSW, one would expect that two cues would elicit an NSW component of identical amplitude whether the two cues are displayed laterally or on the vertical midline. We performed such a test on the present dataset, by estimating NSW activity as done by Feldmann‐Wüstefeld (2021), that is, as the average activity recorded at PO7 and PO8 from midline and lateral 2C trials. The significantly larger amplitude for NSW in response to midline than lateral cues (−0.38 μV vs. −0.69 μV, *t*(18) = 2.2, *p* = .039) is not in line with the above hypothesis, and suggests that more work is needed for an in‐depth exploration of the differences between NSW and SPCNb.

In conclusion, we were able to elicit a bilateral SPCN, the SPCNb, analogously to what we did with the bilateral N2pc (N2pcb; Doro et al., 2020; Monnier et al., 2020). We showed that this SPCNb was modulated in the same way as a typical SPCN, namely, showing an increase in amplitude as the number of to‐be‐memorized objects was increased. Comparisons of ERP modulations induced by the position of the to‐be‐remembered items (lateral vs. midline, upper vs. lower hemifield), as well as the number of cues to be memorized, allowed us to distinguish the N2pc/N2pcb from the SPCN/SPCNb. Because SPCN/SPCNb amplitude was not reduced to nil nor reversed in polarity when objects to remember were displayed in the upper visual field, contrary to the N2pc/N2pcb, our results are more compatible with models positing these two components have partially overlapping albeit distinct neural sources.

## AUTHOR CONTRIBUTIONS

**Yanzhang Chen:** Data curation; formal analysis; investigation; methodology; resources; software; visualization. **Sabrina Brigadoi:** Investigation; methodology; project administration; validation; visualization; writing – original draft. **Arianna Schiano Lomoriello:** Formal analysis; methodology; supervision. **Pierre Jolicœur:** Funding acquisition; investigation; methodology; writing – original draft. **Amour Simal:** Formal analysis; investigation; methodology. **Shimin Fu:** Funding acquisition; visualization. **Valentina Baro:** Conceptualization; validation; visualization; writing – original draft. **Roberto Dell'Acqua:** Conceptualization; investigation; methodology; project administration; resources; supervision; writing – original draft.

## Footnotes

^1^
The term “onset” for SPCN is not always easy to define in contralateral‐minus‐ipsilateral ERP waveforms because of the overlap with N2pc. In some experiments, these components appear clearly separable, due to a clear return to the 0 μV baseline interposed between them. In such cases, SPCN onset can be taken as the time at which a negativity begins following the return of N2pc to 0 μV. Sometimes, however, there is no return to an absolute 0 μV level, and SPCN begins while N2pc has apparently not completely vanished. In this case, onset denotes t_0_ of the time‐window chosen for SPCN amplitude quantification.

^2^
Probably, the most notable exception to the insensitivity of N2pc to variations in the number of targets is represented by visual search tasks requiring to count the number of targets in a search display, when the targets’ number varies within the subitizing range, i.e., from 1 to 3. In this specific case, N2pc amplitude has been shown to scale with the number of targets, much like SPCN (e.g., Benavides‐Varela et al., 2018; Mazza & Caramazza, 2011; Pagano & Mazza, 2012).

^3^
Raw ERP components with an onset earlier than N2pc are not discussed in the specific analytical context (but see “3.2.3 N2pcb and SPCNb for upper and lower visual hemifield cues”). Figure 3 in particular indicates a magnified P1 component in ERPs elicited by midline cues compared to lateral cues peaking at about 90 ms, which is almost certainly due to the physical/sensory difference between midline and lateral cue arrays (Hillyard & Picton, 1987; Pratt, 2012).
